# Zinc Signaling in the Mammary Gland: For Better and for Worse

**DOI:** 10.3390/biomedicines9091204

**Published:** 2021-09-12

**Authors:** Moumita Chakraborty, Michal Hershfinkel

**Affiliations:** Department of Physiology and Cell Biology, Faculty of Health Sciences, Ben-Gurion University of the Negev, Beer-Sheva 84105, Israel; hmichal@bgu.ac.il

**Keywords:** zinc, zinc signaling, Zn^2+^ transporters, ZnR/GPR39, mammary gland, breast cancer

## Abstract

Zinc (Zn^2+^) plays an essential role in epithelial physiology. Among its many effects, most prominent is its action to accelerate cell proliferation, thereby modulating wound healing. It also mediates affects in the gastrointestinal system, in the testes, and in secretory organs, including the pancreas, salivary, and prostate glands. On the cellular level, Zn^2+^ is involved in protein folding, DNA, and RNA synthesis, and in the function of numerous enzymes. In the mammary gland, Zn^2+^ accumulation in maternal milk is essential for supporting infant growth during the neonatal period. Importantly, Zn^2+^ signaling also has direct roles in controlling mammary gland development or, alternatively, involution. During breast cancer progression, accumulation or redistribution of Zn^2+^ occurs in the mammary gland, with aberrant Zn^2+^ signaling observed in the malignant cells. Here, we review the current understanding of the role of in Zn^2+^ the mammary gland, and the proteins controlling cellular Zn^2+^ homeostasis and signaling, including Zn^2+^ transporters and the Gq-coupled Zn^2+^ sensing receptor, ZnR/GPR39. Significant advances in our understanding of Zn^2+^ signaling in the normal mammary gland as well as in the context of breast cancer provides new avenues for identification of specific targets for breast cancer therapy.

## 1. Zinc, an Essential Micronutrient

Zinc is a vital trace element present in all body tissues and organs. There are differences in recommendation of expert groups regarding the daily allowance of dietary zinc, with suggested intake of 10–20 mg/day, depending on age and gender [1]. Insufficient nutritional zinc intake increases the risk of zinc deficiency [2,3], and low plasma zinc levels have been recorded in as many as 80% of children in developing countries [4,5]. However, zinc deficiency is not unique in low income countries, as it is frequently found in developed countries as well [2,6], with an estimated 17% of the world’s population at risk of zinc deficiency [5]. Insufficient zinc contributes to the etiology of a wide range of pathologies, including immune system failure, digestive system diseases, wound healing, and cognitive impairment [6,7]. The most severe consequence of zinc deficiency is seen in the rare genetic disorder, acrodermatitis enteropathica (AE), a genetic disorder in which impaired intestinal zinc absorption produces an acute, potentially fatal zinc deficiency [8,9]. Importantly, symptoms of zinc deficiency can typically be reversed by zinc supplementation [10,11]. In neonates and young infants, zinc deficiency is associated with skin lesions, growth retardation and impaired development [12,13]. Surprisingly, symptoms of severe zinc deficiency have been observed in exclusively breast-fed babies [14,15]. These infants exhibited a failure to thrive, which was later determined to be related to defective zinc secretion into milk [16,17,18]. Thus, regulation of zinc homeostasis in the mammary gland is crucial during lactation. Conditioned zinc deficiencies are also known to occur in malabsorption syndromes, chronic liver or renal disease, excessive intake of alcohol, and in certain instances of neoplastic malignancies [19]. Indeed, epidemiological studies have linked dietary zinc deficiency to increased risk of cancer [20,21]. However, a complicated picture of both abnormally high and low serum zinc levels in malignant cells, suggests that zinc may be involved in various aspects of cancer progression.

Zinc ions (Zn^2+^) act as structural components of many enzymes and transcription factors [22]. This important structural function may account for a well-established role of Zn^2+^ in proliferation, and, indeed, Zn^2+^ deficiency is associated with attenuation of cell growth [23,24,25]. Moreover, DNA damage was demonstrated in Zn^2+^-depleted cells, while its supplementation enhanced genome stability even following radiation treatment [26]. Nevertheless, Zn^2+^ has another important role: acting as a signaling molecule. This conclusion is supported by the large number of proteins involved in compartmentalization of the ion, allowing transient local changes in Zn^2+^ concentrations, as required for signaling [27,28,29]. Transient changes in cytoplasmic Zn^2+^ levels and its interaction with cellular kinases or receptors induce what is termed Zn^2+^ signaling. An example of this is the interaction of Zn^2+^ with the growth hormone receptor, and its modulation of the insulin-like growth factor-I (IGF-1) pathway [25]. More recent studies have identified Zn^2+^ as a first messenger, regulating the ZnR/GPR39 G-protein coupled receptor, or as a second messenger, activating major pathways associated with proliferation [30,31,32].

Under physiological conditions, cellular Zn^2+^ is mostly bound to proteins, and “free” Zn^2+^ concentrations are in the picomolar to nanomolar range [33]. Yet, “free” Zn^2+^ ions do occur, and are typically sequestered in cellular vesicles and in organelles, including the Golgi, endoplasmic reticulum, and mitochondria, thereby serving as Zn^2+^ reservoirs [34,35,36]. Regulation of free Zn^2+^ in the cytosol is largely achieved either by binding to metallothioneins (MT) or by Zn^2+^ transporters [27,37,38]. Two protein families are known to mediate Zn^2+^ transport across cellular membranes, as Zrt- and Irt-related proteins (ZIP) mediate influx into the cytoplasm and Zn^2+^ transporters (ZnT) move Zn^2+^ out of the cytoplasm [37,39]. Zn^2+^ homeostasis is tightly regulated by these proteins under normal circumstances. Intriguingly, Zn^2+^ levels are dysregulated in the microenvironment surrounding cancer tissue [29]. This finding correlates with changes in the expression profile of various Zn^2+^ transporters related to the presence of cytokines and growth factors in the tumor’s microenvironment [40].

Zn^2+^ is emerging as an important player in mammary gland function under both physiological and neoplastic conditions. We will discuss molecular mechanisms mediating the activities of Zn^2+^ in this tissue.

## 2. Zn^2+^ as a Signaling Molecule

For Zn^2+^ to act as a signaling molecule, both its intracellular and extracellular levels must transiently change. In addition, there must be protein targets that detect these changes and subsequently trigger downstream effects. On a fast scale, intracellular Zn^2+^ transients involve a Zn^2+^ release from intracellular stores, e.g., endoplasmic reticulum or Zn^2+^-binding metallothioneins, in response to stimuli [32,41,42]. For example, antigen binding in mast cells induces extracellular Ca^2+^ influx that upregulates mitogen-activated kinase (MAPK). This, in turn, activates the release of sequestered Zn^2+^, thereby raising the intracellular Zn^2+^ concentration [32]. Similar rapid changes in intracellular Zn^2+^ levels are produced by its release from stores following epidermal growth factor (EGF) stimulation, which induces casein kinase 2 phosphorylation of a Zn^2+^ transporter, ZIP7 [43]. In addition, activation of the signal transducers and activators of transcription 3, STAT3 pathway functionally modulates Zn^2+^ transporter activity [27,43]. Late Zn^2+^ signaling events, occurring several hours after cell stimulation, and are dependent upon changes in expression of Zn^2+^ transporters that modulate the transfer of Zn^2+^ from one compartment to another [27,37]. Both fast and late types of cytoplasmic Zn^2+^ rises place this ion in the category of classical second messenger, directly regulating major signaling pathways. Among those pathways activated by Zn^2+^ are EGFR [44], the IGFR-1 [45], and MAPK [46]. Changes in cytosolic Zn^2+^ levels can also modulate signaling via inhibition of phosphatases activity [47].

Extracellular Zn^2+^ levels can also transiently increase, as many cell types secrete Zn^2+^ from intracellular vesicles to the extracellular region. Such release is documented in neurons, pituitary cells, prostate epithelial cells, mast cells, granulocytes, Paneth cells in the intestines, pancreatic cells, and mammary gland epithelia [48,49,50,51,52]. The secreted Zn^2+^ can then interact with cell surface proteins that have Zn^2+^-binding modulatory sites, including ion channels, neurotransmitter transporters, and G protein-coupled receptors [53,54,55]. For example, changes in extracellular Zn^2+^ induce transactivation of EGFR in airway epithelial cells [56], as well as activation of the MAPK pathway [57,58]. Thus, extracellular Zn^2+^, acting as a first messenger in mouse embryonic stem cells (ES), induces proliferation through upregulation of PI3K, MAPK, and mTOR signaling pathways [59]. Significantly, a Gq protein-coupled receptor (GPCR), initially called ZnR, is a specific target that distinctly senses extracellular Zn^2+^ and triggers metabotropic calcium (Ca^2+^) release [60]. Later, it was demonstrated that Zn^2+^ is the ligand of the orphan receptor GPR39 [61], and we showed that it mediates metabotropic ZnR activity, which is now termed ZnR/GPR39 [30,62].

## 3. Zn^2+^ and Its Transporters in the Physiology of Mammary Gland

The importance of Zn^2+^ to mammary gland development is manifested by the severe consequences of Zn^2+^ deficiency. Under these conditions, extreme defects in morphology and function, including oxidative stress and inflammation, are described in the non-lactating [63,64] as well as in the lactating gland [65]. During puberty, growth and differentiation of the epithelial glandular structures within the fat pad are driven by the growth hormones, EGF and IGF-1, as well as by the estrogen receptor [66]. Interestingly, all of these pathways are modulated by Zn^2+^, as described above [67]. During pregnancy and lactation, progesterone and prolactin are responsible for growth of the alveolar epithelial structures, and milk production and secretion [68]. At this stage, Zn^2+^ is suggested to regulate proliferation and differentiation of the epithelial cells via its interaction with kinases downstream to the prolactin receptor. Mammary gland involution, following lactation, is also highly dependent on Zn^2+^ that acts as a regulator of apoptosis via mitochondrial or lysosomal pathways [69,70]. The Zn^2+^ transporter, ZnT2, is essential for accumulation of Zn^2+^ in lysosomes and assembly of the vacuolar ATPase for initiation of mammary gland involution [70], via a mechanism only partially understood.

**Figure 1:**
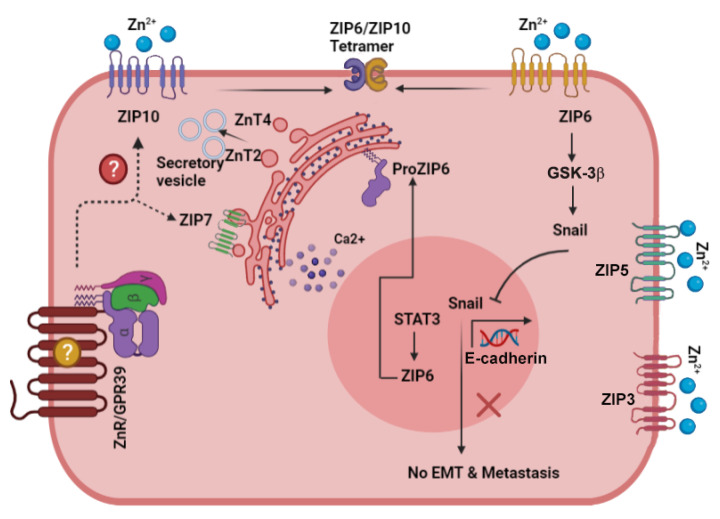
Scheme describing the main signaling pathways activated by Zn^2+^ in the normal mammary gland cells. The Zn^2+^ transporters that play a role in mammary gland physiology and their localization are described in the figure. In particular, note ZnT2, that plays a role in vesicular Zn^2+^ accumulation during lactation and involution, and ZIP3/ZIP5, that were suggested to mediate Zn^2+^ influx. Arrows depict established pathways, while dotted lines and question marks are putative pathways that remain to be explored.

A developing offspring requires relatively large amounts of Zn^2+^ to support its rapid growth and development. Mammary cells are tasked with providing this important nutrient, which they do at a rate of about 1 mg Zn^2+^ per day during lactation [12,14,71]. Failure of this process results in severe Zn^2+^ deficiency to the infant, leading to, among other things, growth retardation and skin lesions [18,72]. Even under conditions of moderate Zn^2+^ deficiency, diminished cognitive development is observed [73,74]. Indeed, Zn^2+^ is found in secretory vesicles in the mammary gland and its loss is associated with a condition termed neonatal Zn^2+^ deficiency [15]. During lactation, import of Zn^2+^ from the circulation into the mammary cells is therefore crucial, and its concentration in milk is maintained over a wide range of maternal dietary Zn^2+^ intake [75,76,77,78]. In agreement with the strict requirements for Zn^2+^ in the ingested milk, several Zn^2+^ transporters are present in the mammary tissue (Figure 1), providing the means to adjust the levels of this ion and its distribution into the appropriate cellular compartments [71,79,80]. Most prominent is ZnT2, which is responsible for the transport of Zn^2+^ into the secretory vesicles in mammary gland epithelia during lactation [81]. A point mutation in ZnT2 is associated with a dramatic and injurious decrease in levels of Zn^2+^ in the milk [82]. Additional studies have identified other mutations in the gene encoding ZnT2 which result in severely Zn^2+^-deficient breast-fed infants [17,82,83]. ZnT2 has also been linked to the function of mammary epithelial cells during all developmental stages [70,81,84]. During pregnancy, in preparation for lactation, ZnT2 is essential for development of alveolar structures and is linked to regulation of cell polarity and formation of acidic secretory vesicles through recruitment of the vacuolar ATPase [84]. Subsequently, during lactation, expression of ZnT2 on the secretory vesicles is responsible for transport of Zn^2+^ into these milk-containing vesicles [81]. Lactogenic hormones, prolactin and glucocorticoids, regulate ZnT2 expression via activation of kinase signaling cascades [85]. Studies in pancreatic cells, however, indicate that Zn^2+^ itself can transcriptionally activate ZnT2 expression via activation of the metal transcription factor, MTF-1 [85,86]. Following cessation of lactation, the mammary gland undergoes involution, which is also associated with Zn^2+^ transporters. An initial stage in this process is relocation of ZnT2 from late endosomes into lysosomes [70,87,88]. Subsequent interaction of ZnT2 with the vacuolar ATPases plays an important role in lysosomal-induced cell death [84,88]. Interestingly, Zn^2+^ is also required by matrix metalloproteinases (MMPs), which degrade extracellular matrix components and are essential for remodeling of the tissue during pregnancy and involution [28]. The Zn^2+^ transporters, ZnT5 and ZnT6, are expressed on the Golgi apparatus and play a prominent role in activation of Zn^2+^-binding enzymes, such as the tissue non-specific alkaline phosphatase (TNAP) [89,90]. Interestingly, these transporters are also associated with Zn^2+^-deficient maternal milk, as well as low TNAP activity in mothers of severely symptomatic neonates [91].

To accumulate cytoplasmic Zn^2+^, several Zn^2+^ importers from the ZIP family have been identified in the mammary epithelium (Figure 1) [67]. Expression of ZIP5 on the surface of epithelial mammary cells suggested that this transporter is involved in Zn^2+^ uptake [67]. Surprisingly, ZIP3 mediates Zn^2+^ reuptake from the secreted milk within the alveolar lumen [79]. Release of Zn^2+^ from intracellular stores and regulation of proliferation are associated with phosphorylation of the Golgi Zn^2+^ transporter ZIP7, following activation of EGF cascade [43]. It remains to be seen how other ZnTs and ZIPs that are expressed on mammary epithelial cells function to maintain Zn^2+^ homeostasis and normal function of the mammary gland.

## 4. Zn^2+^ Homeostasis and Zn^2+^ Transporters in Breast Cancer

Considering the well-established roles for Zn^2+^ in cell proliferation and survival, it is not surprising that this ion also participates in cancer progression [25,92]. However, because Zn^2+^ modulates immune responses, it may also be considered as an anti-cancer agent, for example via regulation of anti-tumor activity by T-lymphocytes [93,94,95]. Loss of intracellular Zn^2+^ in prostate tumor epithelial cells is associated with downregulation of the transporter ZIP1 and tumor progression via enhanced cell proliferation [96,97]. Thus, it would appear likely that correcting Zn^2+^ deficiency will attenuate prostate cancer progression. However, treatment of cancerous prostate tumors by elevating dietary or serum Zn^2+^, which does not directly affect the “free” Zn^2+^ content of prostate cells, proved controversial, and more specific tools were sought [98,99]. In breast cancer patients, measurement of total Zn^2+^ in the serum or within the malignant cells demonstrated abnormal concentrations, suggesting the involvement of Zn^2+^ dysregulation in progression of this malignancy as well [100,101]. While serum Zn^2+^ levels are reduced in most cancers, breast and lung tumor tissues have elevated levels of “free” Zn^2+^ when compared to the normal tissue [102,103]. Tissue Zn^2+^ accumulation, moreover, is dependent on breast cancer subtype, suggesting this could serve to define the molecular subtype of the cancer and predict its response to therapy [103,104]. Analyses of isotopic composition of Zn^2+^ in breast tumors, relative to normal mammary tissue, determined that Zn^2+^ is mostly bound to sulfur-rich proteins, likely metallothioneins in the malignant tissue [105,106]. Such analyses suggest a novel approach for using Zn^2+^ as a biomarker to identify the tumor subtype and prognosis [107].

Changes in cytoplasmic and extracellular “free” Zn^2+^ can affect tumor progression via multiple pathways. Cytoplasmic “free” Zn^2+^ may rise in the context of oxidation/reduction reactions that liberate Zn^2+^ from metallothioneins [38,108,109] and are very common in tumor environments. This Zn^2+^ can then modulate phosphatases and kinases associated with signaling pathways involved in cell proliferation and metastasis [47,90]. In addition, increased cytoplasmic “free” Zn^2+^ can alter the expression of various Zn^2+^ transporters that are regulated, either directly via glycosylation [110] or via the Zn^2+^ sensing metal transcription factor MTF-1 [111]. Changes in expression of Zn^2+^ transporters may, in turn, increase efflux of Zn^2+^ [112] or affect the vesicular concentration of this ion which is available for release by exocytosis. Moreover, hypoxic conditions and tumor necrosis can also trigger Zn^2+^ release from the injured cells [113]. Such changes in extracellular “free” Zn^2+^ levels can regulate matrix metalloproteinases [114,115], which further degrade the extracellular matrix and allow migration (metastasis) of tumor cells. Changes in extracellular Zn^2+^ have also been shown to activate the G-protein-coupled receptor, ZnR/GPR39, and to promote signaling leading to epithelial repair [30,113,116,117]. Only some of these pathways have been studied in breast cancer tissue, and further analysis may provide new and discrete therapeutic targets.

## 5. ZnT Family and Breast Cancer

**Figure 2:**
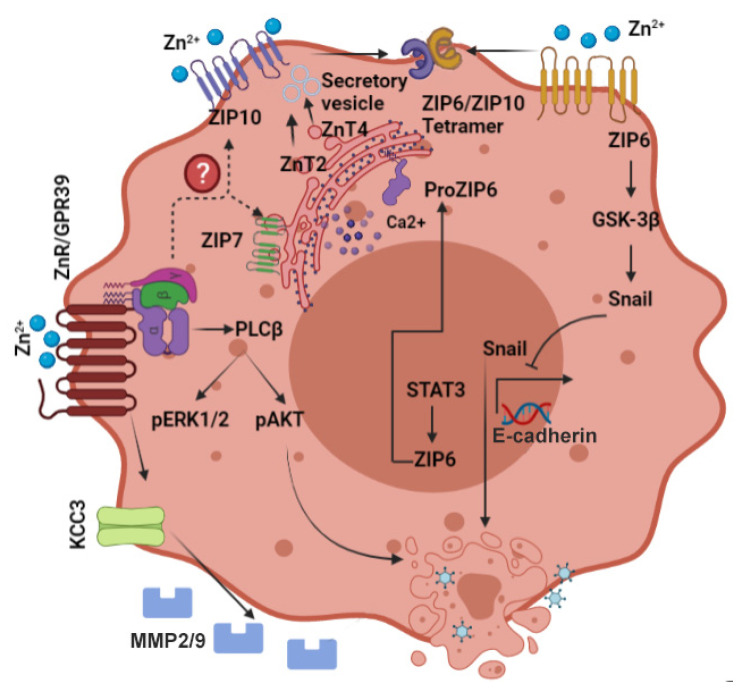
Scheme describing the activation of signaling pathways involved in breast cancer progression and metastasis. Zn^2+^ dyshomeostasis results in activation of multiple pathways that affect cell morphology, migration, and proliferation. Note specifically ZIP10 and ZIP6 that interact to regulate epithelial mesenchymal transition, and ZnR/GPR39 that induces membrane protrusions and MMP2/9 release. Arrows depict established pathways, while dotted lines and question marks are putative pathways for regulation and interaction.

A comprehensive analysis of the distribution of Zn^2+^ transporters in breast cancer tissue and cell lines revealed that transporter protein levels are aberrant [103]. Significant differences in expression of ZnT5, ZnT6, ZnT8, and ZnT9, between basal-estrogen receptor negative (ER-) and luminal-ER positive (ER+) subtypes of breast cancer were demonstrated [103], though how they affect cellular signaling or breast cancer progression remains unclear (Figure 2).

### ZnT2

Studies intended to show how Zn^2+^ accumulation is regulated in tumor cells have largely focused on ZnT2, which has a well-described role in the normal mammary gland (see Section 3). In one such study, ZnT2 was overexpressed in an ER+ breast cancer cell line (T47D) where it appeared to enhance vesicular Zn^2+^ levels in these cells [118]. Knockdown of this transporter increased cytosolic, i.e., “free” Zn^2+^, as well as autophagic cell death, suggesting that, by accumulating Zn^2+^ in vesicles/reducing cytosolic Zn^2+^, ZnT2 reduces cell death and enhances survival of the malignant cells [118]. In addition, increased ZnT2 expression levels in an ER breast cancer cell model induced lysosomal Zn^2+^ accumulation and reduced MMP-2 activity, leading to a decrease in invasive properties of the cells [119]. When breast cancer tumor biopsies were studied, ZnT2 overexpression was demonstrated in luminal (ER+) breast tumors compared to its level in basal (ER-) tumors, and corresponding cell line models [103]. Indeed, in MDA-MB-231 cells, representing basal tumors, overexpression of ZnT2 increased vesicular Zn^2+^ accumulation and decreased their invasiveness [103]. It will be interesting to monitor whether the well-described mutations in ZnT2, which are associated with low levels of Zn^2+^ secretion during lactation, are also linked to specific breast cancer subtypes. In addition, dimerization of ZnT transporters was suggested to provide anther pathway for their localization and functional regulation [120]. Changes in the ZnT2 expression pattern in breast cancer cells may provide a key to modulating Zn^2+^ homeostasis in this tumor. However, it should be noted that such changes may affect expression patterns of other ZnT family members, and this should be carefully addressed.

## 6. ZIP Family and Breast Cancer

The LIV-1 subfamily of ZIP proteins was initially characterized as estrogen-sensitive proteins in breast cancer cells (Figure 2), and specifically associated with ER+ breast cancer, and with metastatic spread of breast cancer tumors [121,122]. Members of the LIV-1 family include ZIP4–8 and ZIP10, all of whom contain a histidine-rich sequence on transmembrane domain 5 that is associated with their Zn^2+^ transport activity [123,124].

### 6.1. ZIP6

The ZIP6 protein, suggested to mediate cytoplasmic Zn^2+^ uptake, was first identified in ER+ breast tumors and is considered as an indicator of this type of cancer [125,126,127]. Expression of ZIP6 is observed primarily in metastases to lymph nodes [128]. Interestingly, ZIP6 is a downstream target of the transcription factor, i.e., signal transducer and activator of transcription 3 (STAT3), which is involved in epithelial to mesenchymal transition (EMT) during development of zebrafish embryos [129]. In breast cancer cells, activation of ZIP6 occurs by its N-terminal cleavage, which induces its translocation to the plasma membrane, triggering cytoplasmic Zn^2+^ influx. This “free” Zn^2+^ rise then activates nuclear localization of the transcription factor, Snail, likely by inhibition of glycogen synthase kinase, GSK-3β, and by reduced expression of the junctional protein, E-cadherin [130]. Repression of E-cadherin expression reduces cell adhesion and induces EMT, a hallmark of invasive cancer. Indeed, silencing ZIP6 in HeLa cervical cancer cells led to reduced cell invasion and dysregulation of the Snail pathway [131].

### 6.2. ZIP7

ZIP7 is localized to the endoplasmic reticulum and is responsible for Zn^2+^ release from intracellular stores [132]. This transporter is regulated by phosphorylation of casein kinase 2 which induces an increase in cellular Zn^2+^ and tyrosine kinase activation [133]. Aberrant signaling triggered by ZIP7 in hormone-resistant breast cancer cells and changes in its expression level in breast tumor tissues have strongly linked this transporter to breast cancer development [134,135]. ZIP7 induces a cytoplasmic Zn^2+^ rise that activates major signaling cascades such as c-SRC, EGFR, and MAPK, and thereby enhances invasion of these cells [135]. Numerous therapeutic approaches are aimed to attenuate tyrosine kinase pathways that play a major role in cancer progression [136,137]. In this way, ZIP7 can serve as a target for regulation of tyrosine kinase pathways. Moreover, protein kinase B/AKT that is constitutively activated in breast cancer cells [138] is also directly activated by ZIP7-mediated Zn^2+^ release in hormone-resistant cells [139]. In a tamoxifen-resistant MCF-7-derived breast cancer cell model (TamR), increased expression of ZIP7 leads to a more aggressive phenotype than the original MCF-7 cells [135]. Removal of ZIP7 from these TamR cells inhibits EGFR and IGF-1R [135], both of which are known to trigger growth of these cells [140]. Significantly, analysis of breast cancer biopsies showed that ZIP7 was positively linked with the proliferation marker Ki67, and was increased in breast cancer samples with metastases to lymph nodes [122,132].

### 6.3. ZIP10

Expression of ZIP10 mRNA is increased in breast cancer cell lines, while its reduction (by knockdown) attenuated migration of these cells [141]. Corresponding elevated levels of ZIP10 mRNA were detected in lymph node metastases of breast cancer biopsies [141]. Several Zn^2+^ transporters have been shown to act as heterodimers, and their interaction is associated with synergistic function. Among these, most prominent is the interaction between ZIP10 and ZIP6 [142]. Heterodimerization of ZIP6 and ZIP10 synergistically enhanced glucose-dependent breast cancer cell migration [143], which could be associated with increased breast cancer mortality in diabetic patients [144]. Knockdown of ZIP10 results in morphological changes in breast cancer cells, which are followed with loss of adhesion and enhanced proliferation of the cells [142]. An elegant recent study has shown that surface expression of ZIP10 and ZIP6 heterodimers is upregulated during mitosis [145]. The Zn^2+^ influx mediated by these transporters drives serine phosphorylation of STAT3 by an unknown mechanism, which then mediates microtubule re-organization and mitosis [145].

## 7. The Zinc Sensing Receptor ZnR/GPR39 and Cancer

The selective Zn^2+^-sensing Gq-protein-coupled receptor (ZnR/GPR39) acts as a link between extracellular Zn^2+^ and intracellular pathways that regulate cell proliferation and survival [30,146]. The ZnR/GPR39 is a member of the rhodopsin-like family, positioned on chromosome 2, q21.2 that encodes two splice variants: GPR39-1a, a full-length receptor of 435 amino acids via two exons 52 kilo Dalton (kDa) in size, and GPR39-1b, which is encoded by the first exon alone (1-285), resulting in a 32 kDa protein that is non-functional [147]. The first exon contains five transmembrane domains (TM 1–5) and the second contains two TM domains (TM 6–7). It was shown that the full-length protein is activated by extracellular Zn^2+^ and mediates metabotropic Ca^2+^ signaling via the IP3 pathway in colonocytes, keratinocytes, and neurons [62,113,116,148,149]. Extracellular Zn^2+^ interacts with ZnR/GPR39 and initiates cell signaling events that trigger release of intracellular Ca^2+^ [60,61,150].

Downstream to the Ca^2+^ signaling, ZnR/GPR39 activates MAPK via extracellular signal-regulated kinase (ERK1/2), and the phosphoinositide-3 (PI3)-kinase pathway [148], both closely linked to cell growth and proliferation [151]. Indeed, cell proliferation and migration, mediated by ZnR/GPR39 in a Zn^2+^-dependent manner, accelerated wound closure in HaCaT keratinocytes [113]. A similar effect on colon epithelial cells was observed in a model of colitis, where the recovery of colon tissue in mice lacking ZnR/GPR39 was impaired [152]. However, BrdU staining indicated that baseline proliferation rates were not significantly different between wildtype and ZnR/GPR39 knockout colon epithelial cells. In prostate cancer cells, ZnR/GPR39 was also associated with upregulation of ERK1/2 and PI3K phosphorylation, and enhanced cell growth [149]. Interestingly, cytoplasmic Zn^2+^ levels in the prostate are high [97], and expression of the ZnR/GPR39 in non-cancer cells was very low, likely due to desensitization of the receptor. In prostate cancer, however, when the Zn^2+^ concentration is decreased [96,97], ZnR/GPR39 may be re-sensitized, such that extracellular Zn^2+^ binding to it will activate Ca^2+^ signaling, leading to proliferation. Further evidence for the role of ZnR/GPR39 in cancer comes from a study of esophageal squamous cell carcinoma (ESCC) shows that overexpression of GPR39 is associated with lymph node metastasis [153]. In contrast, silencing of GPR39 significantly reduced mobility of the ESCC cells. Recently, our lab has determined that ZnR/GPR39 is present in breast cancer cells, and upon activation by extracellular Zn^2+^, increases cell proliferation [154]. Furthermore, consistent with a role for ZnR/GPR39 in breast cancer progression, we demonstrated an increase in expression of the receptor in biopsies from higher grade mammary tumors [154].

More evidence of a role for ZnR/GPR39 activation in cancer progression comes from work showing that it regulates several important ion transporters thought to play a role in cell survival and proliferation. In neurons and colonocytes, activation of ZnR/GPR39 enhanced H^+^ transport by the Na^+^/H^+^ exchanger (NHE) [116,155]. ZnR/GPR39 upregulation of NHE was also seen in colon epithelial cells from wild type, but not ZnR/GPR39 knockout mice [156]. Furthermore, inhibition of PI3K pathways in HT29 colonocytes, which exhibit ZnR/GPR39 signaling, resulted in reversal of the effect of Zn^2+^ on NHE activity and attenuation of the recovery from acidification [116]. In keratinocytes, upregulation of NHE by the ZnR/GPR39 signaling cascade resulted in more rapid epithelial wound repair [113]. Upregulation of NHE activity via ZnR/GPR39 signaling was also observed in mouse hippocampal neurons, leading to recovery of intercellular pH [155], which helps to promote neuronal survival [157]. It has been hypothesized that upregulation of NHE activity may lead to tumor cell proliferation by enhancing aerobic glycolysis [158]. Moreover, elevated NHE activity is correlated with increased cellular pH and decreased extracellular pH. Lowering extracellular pH in tumors is a known trigger for metastasis [159]. In addition to NHE1 stimulation of Na^+^/H^+^ exchange, it also acts as a scaffold protein by recruiting cytoskeletal linker proteins, PI3K and AKT, thereby triggering cell growth [160,161]. It is thought that this may actually be the mechanism mediating the effects of Zn^2+^ and ZnR/GPR39 in cell proliferation and migration.

In hippocampal CA3 neurons, ZnR/GPR39 increases surface expression and activation of K^+^/Cl^−^ -cotransport (KCC) isoform 2 [162,163]. KCC2 has a role in regulating the Cl^−^ gradient and, thereby, GABA_A_ currents in neurons [164]. Similar ZnR/GPR39-dependent upregulation of KCC1 was demonstrated in colonocytes [165] and resulted in enhanced Cl^−^ absorption and decreased water loss in a diarrhea model. Other KCC family members are essential for regulating epithelial cell volume, a function linked to morphological changes underlying formation of plasma membrane protrusions involved in metastasis as well as cell proliferation [166]. Studies in breast cancer cells show that KCC3 is upregulated by ZnR/GPR39 in the ER+ cell lines [167]. A recent study reports that ZnR/GPR39 activation in breast cancer cells leads to activation of K^+^/Cl^-^ cotransporter KCC3, which may locally affect cell volume resulting in formation of protrusions [53]. In addition, ZnR/GPR39 triggers release of matrix metalloproteases, MMP-2 and MMP-9 [53], which degrade the extracellular matrix and facilitate migration. This pathway is essential for enhancing invasion of breast cancer cells through Matrigel and scratch closure. These results indicate that ZnR/GPR39 may be a novel therapeutic target for controlling breast tumor growth and progression.

## 8. Emerging Targets for Breast Cancer Treatment

Development of the normal breast is mostly regulated by estrogen acting through the ER [168], thereby controlling a variety of functions, including cell proliferation, angiogenesis, and apoptosis [66]. The ER signaling is used by breast cancer cells and, in the initial stages of the disease, serves as a major, estrogen-driven, survival pathway [168,169,170]. Therefore, expression of ER is used as a biomarker to guide therapy in breast cancer [171]. As a first line, ER-expressing (positive) breast cancer patients are generally treated with antihormones such as tamoxifen [169,172]. Electron microscope analysis showed that tamoxifen treatment of breast cancer cells induced apoptosis or autophagy, with some cells displaying signs of both [173,174]. While this treatment is initially highly effective in attenuating growth, resistance of tumors to tamoxifen gradually develops usually in all patients, leading to intermittence and re-appearance of the disease [175,176]. Tamoxifen resistance may occur through modification of different signaling pathways involving growth factors such as EGF, IGF receptors, and the HER2 tyrosine kinase receptor [77,170,172]. Even though detection and treatment of metastatic breast cancer have advanced in recent years, the mortality caused by this disease remains high, due principally to such therapy-resistant breast cancer cells [169,177,178]. Therapy-resistant breast cancer can be intrinsic, which accounts for approximately 15–20% of ER-positive breast cancer, or acquired, which accounts for an additional 25–30%, and involves the loss of the hormonal effects [179,180]. A more aggressive type of breast cancer is characterized by the absence of all hormone receptors and is referred to as triple negative breast cancer, or TNBC. This type of tumor is defined by negative expression of ER, progesterone receptor (PR), and human epidermal growth factor receptor-2 (HER2) proteins [181]. TNBC has a higher rate of frequency and shorter survival in the metastatic setting compared to other subtypes of breast cancer. The TNBC cells show a large heterogeneity, and have been divided into several subtypes, presenting unique molecular signatures. Many of these involve mutations in P53 or BRCA genes [171]. Recent and novel therapeutic approaches are aimed at specific checkpoints in the cell cycle, but a lack of biomarkers in TNBC means that chemotherapy remains the first line of treatment. The emergence of immune therapy has led to the targeting of the highly expressed programmed death-ligand 1 (PD-L1) checkpoint protein and to the large numbers of lymphocytes in a subset of TNBC tumors [182]. Identification of novel targets on TNBC tumor cells that can be modulated to reduce proliferation and invasiveness is an as-yet-unmet clinical need. Previous reports suggested specific proteins, such as ER, P53, or of more general targets including GPCRs, which are overexpressed in cancer cells compared to normal tissue [183,184]. As such, ZnR/GPR39, which was shown previously to be overexpressed in breast cancer tissue [154] may prove to be a convenient and effective target. In ER breast cancer cell lines, ZnR/GPR39 activation increased MAPK and PI3K phosphorylation [154]. Thus, ZnR/GPR39 may provide an alternative trigger to MAPK and PI3K/mTOR pathway in ER cells, thereby increasing their cell growth. In agreement with this, increased expression of ZnR/GPR39 was associated with more aggressive phenotypes in breast cancer biopsies [154]. Considering the well-known ability of the pharmaceutical industry to identify modulators of GPCRs, future identification of specific agonists or antagonists that interact with ZnR/GPR39 seems assured, and may well prove an important therapy in and of itself, or in combination with immunotherapy. Such molecular modulators are likely to be at least useful as adjunct therapies given the sheer number of proteins modulated by Zn^2+^, among them are the Zn^2+^ transporters discussed above. Thus, a modulator of ZnR/GPR39 that will not trigger other Zn^2+^-dependent pathways is of clear importance.

## 9. Summary and Conclusions

Zinc is an essential ion, required for cell function and proliferation. Less appreciated is its role in promoting oncogenesis and in metastasis. The important role of this ion in itself and of the transporters that are involved in maintaining cellular Zn^2+^ homeostasis suggest that they may provide novel targets for cancer therapies. In addition, our work suggests that ZnR/GPR39 can activate critical compensatory pathways following the loss of hormone regulation of mammary cell growth. Thus, ZnR/GPR39 represents a highly viable target for new molecular or immune approaches to hormone-resistant cancers and TNBC.
